# Two case reports of fulminant giant cell myocarditis treated with rabbit anti-thymocyte globulin

**DOI:** 10.1093/ehjcr/ytae128

**Published:** 2024-03-14

**Authors:** Colin Bartz-Overman, Sarah Li, Balaram Puligandla, Nalini Colaco, Johannes Steiner, Luke Masha

**Affiliations:** Department of Medicine, Oregon Health & Science University, Portland, OR, USA; Department of Medicine, Oregon Health & Science University, Portland, OR, USA; Department of Pathology and Laboratory Medicine, Oregon Health & Science University, Portland, OR, USA; Knight Cardiovascular Institute, Oregon Health & Science University, 3303 S. Bond Avenue, Portland, OR 97239, USA; Knight Cardiovascular Institute, Oregon Health & Science University, 3303 S. Bond Avenue, Portland, OR 97239, USA; Knight Cardiovascular Institute, Oregon Health & Science University, 3303 S. Bond Avenue, Portland, OR 97239, USA

**Keywords:** Giant cell myocarditis, Myocarditis, Rabbit anti-thymocyte globulin, Cardiogenic shock, Cardiac transplant, Case reports, Case series

## Abstract

**Background:**

Giant cell myocarditis (GCM) is an inflammatory form of acute heart failure with high rates of cardiac transplantation or death. Standard acute treatment includes multi-drug immunosuppressive regimens. There is a small but growing number of case reports utilizing rabbit anti-thymocyte globulin in severe cases.

**Case summary:**

Two cases are presented with similar presentations and clinical courses. Both are middle-aged patients with no significant past medical history, who presented with new acute decompensated heart failure that quickly progressed to cardiogenic shock requiring inotropic and mechanical circulatory support. Both underwent endomyocardial biopsies that diagnosed GCM. Both were treated with a multi-agent immunosuppressive regimen, notably including rabbit anti-thymocyte globulin, with subsequent resolution of shock and recovery of left ventricular ejection fraction. Both remain transplant-free and without ventricular arrhythmias at 7 months and 26 months, respectively.

**Discussion:**

In aggregate, these cases are typical of GCM. They add to growing observational data that upfront rabbit anti-thymocyte globulin may reduce morbidity and mortality in GCM, including potentially preventing the need for complex interventions like orthotopic heart transplantation.

**Learning points**

- Giant cell myocarditis (GCM) requires endomyocardial biopsy for histopathologic diagnosis.
- Standard acute treatment regimens for GCM include multi-drug immunosuppressive regimens with high-dose corticosteroids, calcineurin inhibitors, mycophenolate mofetil, and/or azathioprine.
- There is growing observational evidence, in the form of case reports, that early use of rabbit anti-thymocyte globulin in severe GCM can improve clinical outcomes, including decreasing need for cardiac transplantation.

## Introduction

Giant cell myocarditis (GCM) is an inflammatory form of acute heart failure with high rates of cardiac transplantation or death. It tends to impact young and middle-aged adults without predilection for either sex.^1–3^ A full understanding of the pathogenesis remains elusive, with evidence suggesting possible contributions from viral infections, autoimmune diseases, and genetic triggers.^4^ The histopathologic hallmarks are multinucleated giant cells with inflammatory T-lymphocytes, myocyte necrosis, and eosinophilic infiltrates.^3^ The most common presentation is rapidly progressive congestive heart failure that does not respond to standard therapy, often presenting as ventricular arrhythmias, atrioventricular block, and cardiogenic shock.^2,3^ Diagnosis is challenging and can be delayed by non-specific findings on electrocardiogram (ECG), cardiac biomarkers, and echocardiography. Endomyocardial biopsy is required for diagnosis, although false negative rates are estimated at 15–20%.^5^

Current recommendations for management are based primarily on observational data and expert consensus.^6^ The rare nature of GCM, combined with variable clinical presentation and high acuity, has precluded both large randomized prospective trials and longitudinal registries. Standard medication regimens include multi-drug immunosuppression with high-dose corticosteroids, calcineurin inhibitors, and mycophenolate mofetil (MMF) or azathioprine (AZA).^6^ Despite treatment, GCM often progresses and results in either heart transplantation or death. Kandolin *et al.*^7^ reviewed 32 patients with biopsy-confirmed GCM who were treated with multi-drug immunosuppression and found 1-year and 5-year transplant-free survival rates of 70% and 50%, respectively. Rates of sustained ventricular tachycardia were very high (65%), and persistent severe LV systolic dysfunction following treatment was common (25%).^7^

## Summary figure

Effects of rabbit anti-thymocyte globulin (rATG).^8^

**Figure:**
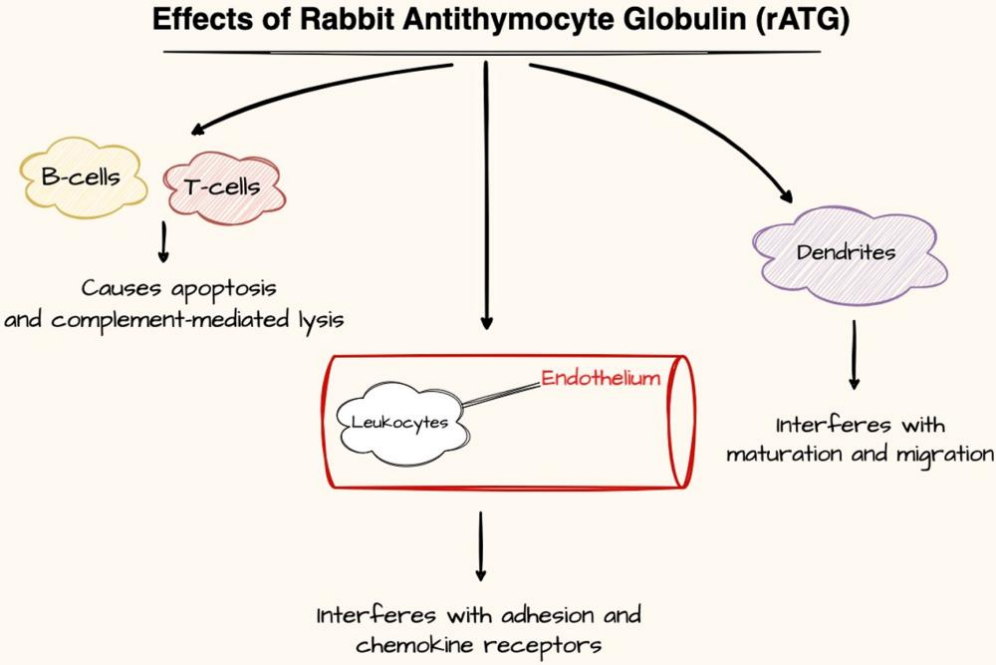


In addition to the aforementioned immunosuppressive agents, there is a small but growing number of case reports utilizing rabbit anti-thymocyte globulin (rATG), a polyclonal antibody known to deplete T-cells, induce apoptosis of B-cells, and interfere with dendritic cell maturation and migration, amongst other immunosuppressive effects.^8^ Historically, this agent has been used in solid organ transplantation and as prophylaxis against graft-vs.-host-disease in stem cell transplantation. In the medical literature, we found nine case reports of rATG use in refractory and fulminant cases of GCM.^9–14^

Here, we present two cases of biopsy-proven fulminant GCM complicated by cardiogenic shock requiring mechanical circulatory support (MCS) with marked improvement following treatment with rATG.

## Case presentations

### Patient 1

**Figure 1:**
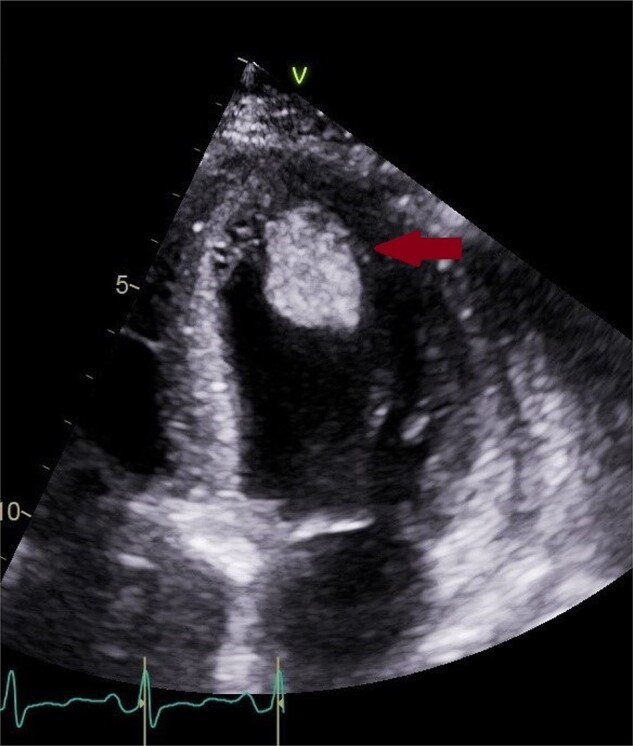
Four-chamber view showing increased wall thickness secondary to tissue oedema and a large mobile left ventricular thrombus, denoted by the arrow (3 × 2 cm). With anticoagulation for 3 months, this thrombus resolved entirely.

**Figure 2:**
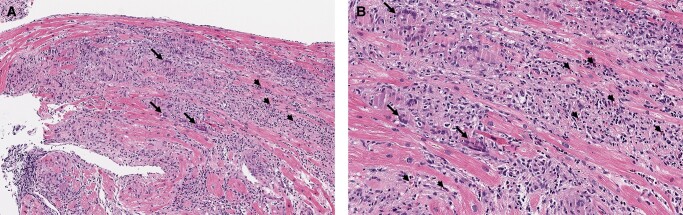
Hematoxylin and eosin stain of endomyocardial biopsy from Patient 1, viewed at ×100 (*A*) and ×200 (*B*). Dense interstitial inflammatory infiltrate containing multinucleated giant cells (arrow), lymphocytes, and eosinophils (arrowhead).

A 62-year-old female with no significant past medical history presented to an outside hospital with dyspnoea and chest heaviness. She was found to have an elevated troponin I of 4.82 ng/mL and a N-terminal pro B-type natriuretic peptide (NT-proBNP) of 7030 pg/mL. Initial ECG showed sinus tachycardia with right axis deviation. Echocardiogram demonstrated left ventricular (LV) ejection fraction (EF) (LVEF) of 50–55% with moderate concentric LV hypertrophy (LVH) and a mild-to-moderate-sized effusion without tamponade. She received diuretics and was discharged with a plan for ischaemic evaluation as an outpatient. Over the next month, she presented multiple times with similar symptoms and was found to have acute kidney injury and an unspecified tachyarrhythmia not captured on ECG. Repeat echocardiogram 11 days later found an LVEF of 25–30% with hypokinesis and stable pericardial effusion. Coronary angiogram found no disease and she was again discharged. She subsequently presented to our hospital and found to be in cardiogenic shock with right heart catheterization showing elevated right- and left-sided pressures with a cardiac index of 1.8 L/min/m^2^ by thermodilution. Her course was complicated by an oliguric acute kidney injury requiring temporary continuous renal replacement therapy and a large ventricular apical thrombi for which she received anticoagulation (*Figure 1*). Screening was negative for human immunodeficiency virus, cytomegalovirus, coronavirus 19, influenza A and B, and hepatitis A, B, and, C. Testing for Epstein–Barr virus was consistent with active or recent infection. Endomyocardial biopsy revealed GCM (*Figure 2*).

Following a multidisciplinary discussion, a decision was made to insert an intra-aortic balloon pump instead of Impella device to minimize the risk of LV thrombus embolization.

**Figure 3:**
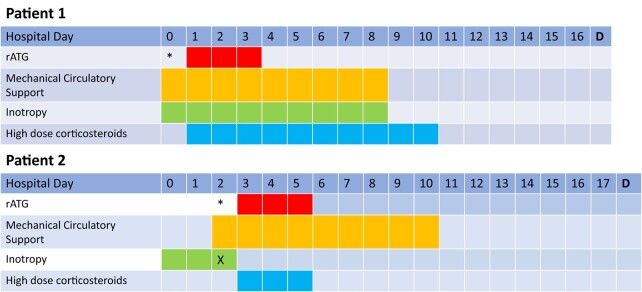
Hospitalization timelines for Patients 1 and 2. Note: Patient 1 initially presented to an outside hospital 23 days prior to admission at our facility. The patient initially presented to an outside hospital 1 day prior to transfer to our facility. ‘*’, Date of histopathologic diagnosis from endomyocardial biopsy. ‘X’, inotropy stopped because of ventricular arrhythmias. ‘D’, Day of discharge.

Dobutamine and epinephrine infusions were started, with the option available to escalate to venoarterial extracorporeal membrane oxygenation (VA-ECMO) if these therapies failed. A cardiac transplant evaluation was initiated. Following histopathologic diagnosis, 100 mg of rATG was administered for 3 consecutive days along with high-dose methylprednisolone. This rATG dosing regimen, used for both patients, was based on prior case reports.^12^ Following rATG administration, she had a mild thrombocytopenia but did not require platelet transfusion. Echocardiogram on the final day of this regimen showed LVEF of <20%. Within 8 days of starting rATG, her haemodynamics normalized, the intra-aortic balloon pump was removed, and inotropy and haemodialysis were discontinued (*Figure 3*). After completion of rATG, she received a prolonged prednisone taper, MMF (1000 mg BID), and tacrolimus dosed to a trough goal of 8–10 ng/mL. Repeat echocardiogram prior to discharge showed LV recovery with an EF of 65–70%. She was discharged on hospital Day 17 with guideline-directed medical therapy of angiotensin receptor/neprilysin inhibitor, beta-blocker, and mineralocorticoid antagonist. She left the hospital with a LifeVest and later underwent placement of single-chamber implantable cardioverter defibrillator (ICD) for primary prevention as the durability of remission with rATG is unknown and GCM has high rates of recurrent ventricular arrhythmias despite treatment.^7^

Positron emission tomography-computed tomography (PET-CT) with fludeoxyglucose F18 1 month after hospitalization was obtained to establish a baseline and showed no abnormal myocardial uptake. She remains free of both significant arrhythmia and cardiac transplant at 7 months of follow-up.

### Patient 2

A 42-year-old male with no significant past medical history presented to an outside hospital with 3 days of viral prodrome including cough, fatigue, joint pain, malaise, and dyspnoea. He was found to have hypoxaemia requiring 4 L supplemental oxygen, leucocytosis [white blood cells (WBC) 17/μL], thrombocytosis (platelets 567/μL), troponin I of 56.5 ng/mL, BNP of 3300 pg/mL, and NT-pro BNP 43 241 pg/mL. Initial ECG showed sinus tachycardia with right bundle branch block. Echocardiography showed severe global hypokinesis with LVEF of 20–25% with LV internal diameter end-diastole of 5.2 cm. Coronary angiogram showed patent coronary arteries. Right heart catheterization found elevated right- and left-sided pressures with a cardiac index of 1.7 L/min/m^2^. He experienced a ventricular tachycardia/fibrillation arrest shortly after presentation and was intubated for 4 days. Serum testing was negative for human immunodeficiency virus, cytomegalovirus, coronavirus-19, influenza A and B, respiratory syncytial virus, Epstein–Barr virus, and hepatitis C. Endomyocardial biopsy showed GCM, with negative testing for cytomegalovirus and Epstein–Barr virus.

For his cardiogenic shock, an Impella 5.5 was placed with background inotropic support and a cardiac transplant evaluation was started. Following diagnosis, he received 100 mg of rATG daily for 3 days along with methylprednisolone. After completion of rATG, he received a prolonged steroid taper, mycophenolate (1000 mg BID), and tacrolimus with a trough goal 6–8 ng/mL. He developed severe thrombocytopenia following rATG administration; however, testing confirmed heparin-induced thrombocytopenia which was treated with bivalirudin. Within 8 days of starting rATG, MCS was able to be discontinued. Repeat echocardiograms on Days 7 and 12 following rATG showed LVEF of 30–35% and 40–45%, respectively. He was discharged on hospital Day 18 with guideline-directed medical therapy of angiotensin receptor/neprilysin inhibitor, beta-blocker, and mineralocorticoid antagonist and a secondary prevention ICD. Four months following initial presentation, he received an alemtuzumab infusion after a baseline PET-CT scan found increased inflammation with elevated troponin I of 6.2 ng/mL in the setting of a normal LVEF. A repeat PET-CT showed reduced inflammation and preserved LVEF, and labs confirmed normalization of the serum troponin. He remains free of both significant arrhythmia and cardiac transplant at 26 months of follow-up.

## Discussion

The cases presented here are typical of GCM. The patients were otherwise healthy, presented with congestive heart failure, and progressed to cardiogenic shock. Both required inotropy and MCS and were evaluated for cardiac transplantation. Treatment with a multi-drug immunosuppressive regimen, including rATG at the time of diagnosis, led to rapid resolution of cardiogenic shock and marked myocardial recovery. Neither suffered clinically significant adverse effects from rATG, the most common of which are leukopoenia and thrombocytopenia. Concerns about these haematological potential complications of rATG did not play a factor in decision-making regarding MCS. Both patients remain transplant-free at the time of manuscript submission, and neither has suffered recurrent ventricular arrhythmias.

Other case reports of GCM treated with rATG have found similar outcomes. Ammirati *et al.*^9^ reported a case of an otherwise healthy 31-year-old male who required inotropy, MCS, and extracorporeal membrane oxygenation, who made a full recovery after receiving immunosuppression which included rATG. Cao *et al.*^10^ published a case initially thought to be cardiac sarcoidosis that was later re-diagnosed as GCM, who was successfully treated with rATG after heart transplantation. Suarez-Barrientos *et al.*^12^ published a case series involving six patients with GCM. Four of the six discharged home without recurrent disease with an average increase in LVEF of 29%. One received a heart transplant and later died from primary graft dysfunction, and one died from bleeding while on MCS. The main complication was thrombocytopenia.

Overall, the two cases reported here add to growing observational data that upfront rATG may reduce morbidity and mortality in GCM, including potentially preventing the need for complex interventions like orthotopic heart transplantation.

## Conclusion

Giant cell myocarditis is a rare cause of progressive cardiomyopathy that, despite treatment advances, has high cardiac transplant and mortality rates. Here, we presented two cases of fulminant GCM with cardiogenic shock that improved following treatment with rATG and remain transplant-free, adding evidence in support of usage in severe disease. Further study is required into the efficacy of rATG and into the durability remission after treatment.

## Data Availability

The data underlying this article are available in the article.

## Lead author biography

Colin Bartz-Overman was born and raised in Yakima, Washington, USA. He majored in political science and later studied medicine at the University of Washington in Seattle. He is completing an Internal Medicine residency at Oregon Health & Science University in Portland, Oregon.

## Notes

**Consent:** Consent was obtained from both Patient 1 and Patient 2, in accordance with the Committee on Publication Ethics (COPE) guidelines.

**Funding:** None declared.
